# Comparison of Beers Criteria and STOPP/START (Screening Tool of Older Persons’ Prescriptions/Screening Tool to Alert to Right Treatment) Criteria for Assessing Inappropriate Drug Use Among Elderly Patients

**DOI:** 10.7759/cureus.102621

**Published:** 2026-01-30

**Authors:** Shrinidhi M R, Ramya Amarnath, Sucheeth Avanti, Bharat Dhareshwar

**Affiliations:** 1 Geriatrics, Mahatma Gandhi Mission's (MGM) Medical College and Hospital, Navi Mumbai, IND

**Keywords:** beers criteria, inappropriate prescription, older patients, start criteria, stopp criteria

## Abstract

Introduction: Prescribing inappropriate drugs is common among older patients due to the presence of multiple comorbidities. Inappropriate drug usage can cause adverse drug reactions, leading to increased risk of hospitalization and mortality. The two most widely used tools among the western population are Beers and STOPP/START (Screening Tool of Older Persons’ Prescriptions/Screening Tool to Alert to Right Treatment) criteria. Comparing these tools among the Indian population will help determine which is more effective in identifying inappropriate drugs and guiding physicians on safer drug usage among elders.

Methods: Geriatric patients aged more than 60 years on at least one chronic medication visiting the OPD or admitted to the ward (n=140) were included in the study. The prescriptions were reviewed with both the Beers and the STOPP/START tool to identify inappropriate medication. Agreement analysis between the two criteria was done using kappa statistics.

Results: The mean age of study participants was 67.8 ± 6.3 years, with 55.7% male patients. Hypertension with cardiovascular disease (73.6%) and diabetes (50.7%) were the most common diseases. Of the total 523 drugs taken by study participants, 61 (11.6%) and 47 (8.9%) drugs had to be stopped based on STOPP and Beer’s criteria, respectively. Using the START criteria, a total of 34 medications had to be added to the existing medication. There was only slight agreement between the two tools (kappa value: 0.093).

Conclusion: The study found a low concordance proportion among the two criteria, suggesting a limited application of these tools among only a specific population of a country in which it was devised. We suggest the need to develop a specific tool to detect inappropriate medication for the Indian population.

## Introduction

The aging population of India is increasing faster than the younger population. The proportion of those aged 60 years and above has risen significantly from 5.3% in 1971 to about 8% in 2020, mainly due to improvements in healthcare, education, and life expectancy [1]. The proportion of the “oldest old,” i.e., individuals above 80 years, has more than doubled from 0.4% of the total population in 1950 to about 0.94% in 2015 and is expected to increase to over 3% by 2050, reaching approximately 48 million older adults [1]. India therefore needs to be prepared to manage geriatric health issues, which differ significantly from those of other population groups.

Inappropriate use of medication in older adults can lead to adverse effects that may outweigh the intended benefits. Hence, identifying such drugs and prescribing safer alternatives is essential. With aging, chronic health conditions become more common, often requiring multiple medications [1,2]. This polypharmacy contributes to drug overuse and increases the risk of drug-drug interactions and associated complications. Older adults are more sensitive to commonly used medications due to altered pharmacokinetics and reduced homeostatic responses. Many developed countries with a higher proportion of geriatric populations have established screening tools to identify inappropriate medications. In India, primary care physicians should play a key role in detecting polypharmacy due to the shortage of geriatric specialists [2]. Knowing which medications are inappropriate and avoiding unnecessary prescriptions are essential practices that all clinicians should adopt [3].

The two most commonly used tools to detect potentially inappropriate medications are the Beers criteria and the STOPP/START (Screening Tool of Older Persons’ Prescriptions/Screening Tool to Alert to Right Treatment) criteria [4]. The Beers criteria, developed by the American Geriatrics Society, are widely used in the United States and focus primarily on identifying inappropriate prescriptions in older adults [4,5]. The STOPP/START criteria, developed in Europe, assess both potentially inappropriate drugs and potential prescribing omissions, guiding clinicians on medications that may need to be added based on patient needs [4,6]. Several international studies [4-7] have compared these tools, while only a few Indian studies have evaluated their application [8]. Comparing these tools in the same patient population will help determine which is more effective in detecting inappropriate medications in the Indian setting and guiding safer prescribing for older adults [9,10].

The Beers criteria were initially developed by Dr. Mark Beers in 1991 and were later updated by the American Geriatrics Society (AGS). Originally designed for nursing home residents, the tool has evolved into a comprehensive guideline applicable to older adults aged 65 years and above [10]. The criteria primarily focus on medications to avoid in older adults, particularly in the presence of specific diseases, drugs to be used with caution, drug-drug interactions, and medications requiring renal dose adjustment. The Beers criteria emphasize situations where risks outweigh benefits; however, a major limitation is that they do not recommend alternative drugs or identify medications that should be initiated. Instead, they primarily assist in identifying prescriptions that warrant review [11].

The STOPP/START tool, developed in 2008 in Europe by expert clinicians and pharmacologists specializing in geriatric medicine, consists of two components. The STOPP component identifies potentially inappropriate prescriptions based on evidence to prevent adverse drug events and drug-drug interactions. The START component identifies omissions of beneficial medications that should be initiated in older adults. The tool categorizes medications by physiological systems, including cardiovascular, respiratory, gastrointestinal, central nervous, musculoskeletal, and endocrine systems [12].

The present study aims to compare the use of these two tools among geriatric patients visiting a tertiary care center, focusing on identifying inappropriate medications and the need for drug modification in the context of polypharmacy. Findings from this study will provide valuable insights for primary care physicians to better detect commonly used inappropriate medications in this age group.

## Materials and methods

Study design and setting

A prospective observational study was conducted among geriatric patients attending the Geriatric Outpatient Department (OPD) or admitted as inpatients at a tertiary care medical college hospital in Navi Mumbai. The study was carried out over a period of three months, from July to September 2025. Both OPD attendees and hospitalized patients were included to ensure representation of varying disease severity and prescribing practices.

Sample size calculation

The sample size was calculated using McNemar’s test formula for paired proportions: n = [ (zα/2 + zβ)² × (p1 + p2) ] / (p1 − p2)², where zα/2 corresponds to a significance level of 0.05, zβ to a power of 80%, p1 to the proportion shifting from positive to negative, and p2 to the proportion shifting from negative to positive. A pilot study conducted among 20 patients estimated the discordant proportions as 25% for p1 and 10% for p2. The minimum sample size calculated was 122; after adding 10% for non-response, the final sample size was 136, which was rounded to 140 participants.

Study population and eligibility criteria

Patients were recruited using predefined inclusion and exclusion criteria. Geriatric patients aged more than 60 years who attended the geriatric OPD or were admitted to the medical wards during the study period and were receiving at least one medication were included. Patients who declined to provide informed consent, those without available prescriptions at the time of evaluation, and terminally ill patients were excluded.

Data collection procedure

After enrolment, the objectives and procedures of the study were explained to each participant, and written informed consent was obtained. Both OPD and inpatient prescriptions were evaluated at a single time point during the study period. Data were collected using a structured and pretested data collection tool comprising three sections. The first section recorded sociodemographic and clinical details, including age, sex, residence, educational status, comorbid conditions, and current medication profile. The second section involved the application of the STOPP/START criteria to identify potentially inappropriate medications and prescribing omissions [11]. The third section applied the American Geriatrics Society Beers Criteria to assess inappropriate drug use among older adults [12]. Both tools were applied independently to each prescription. The STOPP/START criteria and the Beers Criteria are freely available for academic and clinical use.

Statistical analysis

Data entry was performed using Microsoft Excel, and statistical analysis was carried out using IBM SPSS Statistics for Windows, Version 26 (Released 2018; IBM Corp., Armonk, New York, United States). Qualitative variables were expressed as frequencies and percentages, while quantitative variables were summarized using mean and standard deviation. The frequency of inappropriate prescriptions along with their 95% confidence intervals was calculated. Agreement between the STOPP/START criteria and the Beers criteria was assessed using Cohen’s kappa statistic. Kappa values were interpreted as follows: <0 = poor agreement, 0-0.20 = slight agreement, 0.21-0.40 = fair agreement, 0.41-0.60 = moderate agreement, 0.61-0.80 = substantial agreement, and >0.80 = almost perfect agreement.

Ethical considerations

The study was approved by the Institutional Ethics Committee (DHR-EC/2022/SC/12/151). Written informed consent was obtained from all participants prior to their inclusion, and confidentiality of patient information was strictly maintained throughout the study.

## Results

A total of 140 older adults were included in the final analysis, with a mean age of 67.8 ± 6.3 years. More than half of the participants were male and the majority resided in rural areas. The detailed sociodemographic characteristics of the study population are presented in Table 1.

**Table 1:** Sociodemographic details of study participants (N=140) Data presented as frequency and percentage.

Sociodemographic variables		Frequency (%)
Age	60-69 years	73 (52.1)
	70-79 years	55 (39.3)
	>80 years	12 (8.6)
Gender	Male	78 (55.7)
	Female	62 (44.3)
Residence	Rural	83 (59.3)
	Urban	57 (40.7)
Educational status	Illiterate	21 (15.0)
	Primary and middle school	45 (32.1)
	Secondary or high school	49 (35.0)
	Graduate/postgraduate/diploma	25 (17.9)
Occupational status	Working	74 (52.9)
	Not working	66 (47.1)

Hypertension and its related complications were the most common comorbidities, followed by diabetes. The distribution of comorbidities is shown in Table 2.

**Table 2:** Co-morbidities present among study participants (N=140) HT: Hypertension; CVA: Cardiovascular disease; CAD: Coronary artery disease; COPD: Chronic obstructive pulmonary disease. Others include arthritis, Parkinson’s disease, skin disease, and autoimmune disease.

Disease	Frequency (%)
HT/CVA/CAD	103 (73.6)
Diabetes	71 (50.7)
COPD/Asthma	22 (15.7)
Thyroid disorder	15 (10.7)
Psychiatric illness	14 (10.0)
Seizure	5 (3.5)
Cirrhosis	4 (2.8)
Others	6 (4.2)

The total number of medications recorded in the study was 523, with a mean intake of 4.7 ± 1.2 drugs per elder. Overall, 11.6% (95% CI: 9.0-14.7%) of medications were identified as inappropriate by the STOPP criteria and 8.9% (95% CI: 6.6-11.5%) by the Beers Criteria. The system-wise distribution of drugs that needed to be stopped under both criteria is presented in Table 3.

**Table 3:** Summary of drugs stopped under a system based on the STOPP/START medication review tool (n=71) Data presented as frequency and percentage. Beers Criteria as per the American Geriatrics Society [11]; STOPP/START criteria were applied as per O’Mahony et al. [12]. STOPP/START: Screening Tool of Older Persons’ Prescriptions/Screening Tool to Alert to Right Treatment

System	STOPP -START (N,%)	Beers criteria (N,%)
Cardiovascular	25 (40.9)	11 (23.4)
Central Nervous System	11 (18.0)	8 (17.0)
Gastrointestinal	1 (1.6)	13 (27.6)
Chest	8 (13.1)	2 (4.2)
Musculoskeletal	10 (16.3)	7 (14.8)
Urogenital	3 (4.9)	2 (4.2)
Endocrine	2 (3.2)	1 (2.1)
Falling/syncope	1 (1.6)	3 (6.4)
Total	61 (100)	47 (100)

Using the START criteria, 34 medications were identified as necessary additions to the patients’ existing prescriptions. The distribution of these drugs is shown in Table 4.

**Table 4:** Drugs to be started based on STOPP/START criteria Data presented as frequency and percentage. Drug initiation assessed using START criteria as described by O’Mahony et al. [12]. STOPP/START: Screening Tool of Older Persons’ Prescriptions/Screening Tool to Alert to Right Treatment

Initiation of drugs	Frequency (%)
Cardiovascular	7 (20.5)
Chest	1 (2.9)
Central Nervous System	2 (5.8)
Gastrointestinal	5 (14.7)
Musculoskeletal	12 (35.2)
Endocrine	7 (20.5)
Total	34 (100)

Among the drugs requiring discontinuation based on STOPP/START criteria, calcium channel blockers used for hypertension were the most common (14/61), primarily due to chronic constipation, followed by systemic corticosteroids for chronic obstructive airway disease (7/61) and long-term risperidone use exceeding one month (6/61). According to the Beers criteria, proton pump inhibitors (11/47) and nifedipine (8/47) were the most frequently inappropriate medications (Table 5).

**Table 5:** Most common inappropriate medications identified by STOPP/START and Beers criteria Data presented as frequency. STOPP/START criteria applied as per O’Mahony et al. [12] and Beers criteria as per the American Geriatrics Society [11]. STOPP/START: Screening Tool of Older Persons’ Prescriptions/Screening Tool to Alert to Right Treatment

Tool	Drug	Indication	Frequency (n)	Reason for inappropriateness
STOPP	Calcium channel blockers	Hypertension	14	Associated with chronic constipation
STOPP	Systemic corticosteroids	COPD/Asthma	7	Long-term use without clear indication
STOPP	Risperidone (>1 month)	Psychiatric illness	6	Increased risk of falls and extrapyramidal effects
Beers	Proton pump inhibitors (>8 weeks)	Acid peptic disease	11	Increased risk of Clostridioides difficile infection and fractures
Beers	Nifedipine	Hypertension	8	Risk of hypotension and falls

Agreement analysis using Cohen’s kappa showed slight agreement between the Beers and STOPP/START criteria, as shown in Table 6.

**Table 6:** Concordance of inappropriate drugs between STOPP/START and Beers criteria Agreement assessed between Beers Criteria [11] and STOPP/START criteria [12] using Cohen’s kappa statistic. Kappa values were interpreted as follows: <0 = poor agreement, 0–0.20 = slight agreement, 0.21–0.40 = fair agreement, 0.41–0.60 = moderate agreement, 0.61–0.80 = substantial agreement, and >0.80 = almost perfect agreement. STOPP/START: Screening Tool of Older Persons’ Prescriptions/Screening Tool to Alert to Right Treatment

Beers criteria	STOPP/START		Kappa (95% CI)
	Yes (n=61)	No (n=462)	
Yes (n=47)	10	37	0.093 (-0.012- 0.199)
No (n=476)	51	425	

## Discussion

The presence of multiple comorbidities among older adults has increased with rising life expectancy. This has led to polypharmacy for disease management, consequently increasing the risk of drug-drug interactions, drug-disease interactions, adverse drug effects, and poor medication adherence [9]. The present study aimed to estimate the burden of inappropriate medication use among elders visiting a tertiary care hospital and to compare two widely used frameworks: the Beers criteria and the STOPP/START criteria.

In the present study, 61 (11.6%) and 47 (8.9%) medications, respectively, were identified for discontinuation using the STOPP/START and Beers criteria from a total of 523 drugs taken by 140 elders. This indicates that the STOPP/START tool detected more inappropriate medications than the Beers Criteria.

A study from Spain involving 81 hospitalized older adults reported inappropriate prescribing in 48% of cases using STOPP and 25% using the Beers Criteria. The authors also identified drug omissions in 44% of patients using the START tool [6]. Similarly, an Indian study from Kerala found inappropriate prescriptions in 61.5% using STOPP/START and in 38.5% using the Beers Criteria among 260 hospitalized older adults [13]. In contrast, studies from Gujarat and Brazil reported higher detection rates with the Beers Criteria (26.3%-51%) compared with STOPP/START (14%-33.8%) [14,15]. A study from Kuwait showed slightly higher detection using STOPP (55.7%) than Beers (53.1%) [16]. Variations in findings across studies may be attributed to differences in prescribing patterns, prevalence of diseases, drug availability, affordability, and the type of healthcare setting (primary vs. tertiary care).

In our study, calcium channel blockers prescribed to hypertensive patients with chronic constipation were the most common inappropriate medications identified using the STOPP/START criteria. For the Beers Criteria, proton pump inhibitors used for more than eight weeks were most frequently identified as inappropriate due to their association with risks such as Clostridioides difficile infection and bone fractures. Similar findings were reported by Khan et al. and Keche et al. in Nepal and Central India, respectively [17,18]. In the Kerala study by Prasanth et al., loop diuretics used as first-line therapy for hypertension were the most common STOPP-listed inappropriate medication, while sliding scale insulin (SSI) was the most common under the Beers Criteria [13]. SSI is considered unsafe in older adults due to an increased risk of hypoglycemia, diabetic complications, and lack of proven benefit.

Based on the START criteria in the present study, calcium and vitamin D supplementation for osteoporosis and statins for diabetics with cardiovascular risk were the most common medications requiring initiation. Other studies have also reported similar findings [6,18].

The agreement between the two tools in our study was very low, with a kappa value of 0.09. Khan et al. also observed poor agreement (κ = 0.102) between the two frameworks [17]. The low concordance suggests limitations in applying these tools beyond the populations for which they were originally designed. This highlights the need for developing country-specific tools to identify inappropriate medications more accurately. Enhancing awareness among physicians about commonly inappropriate medications for older adults is essential to support safer prescribing practices. Optimizing drug therapy in older adults requires evidence-based tools, multidisciplinary collaboration, continuing medical education, and a comprehensive understanding of the interplay between polypharmacy, multimorbidity, and individual patient preferences.

Limitations

This study has certain limitations. Being a single-center study conducted at a tertiary care hospital, the findings may not be generalizable to all healthcare settings, particularly primary care and rural hospitals. The analysis pooled outpatient and inpatient prescriptions due to sample size constraints, and separate subgroup analysis for these settings could not be performed. Functional status and activity level of participants were not formally assessed, which may have influenced prescribing decisions and the identification of omissions under the START criteria. In addition, clinical outcomes such as adverse drug reactions, hospital readmissions, or mortality were not evaluated. Future multicenter studies with larger sample sizes, stratified analyses by care setting, and assessment of functional status are needed to develop and validate context-specific tools for identifying inappropriate prescribing in the Indian geriatric population.

## Conclusions

In this study of geriatric patients attending a tertiary care center, the STOPP/START criteria identified a higher proportion of potentially inappropriate medications compared with the Beers Criteria and additionally detected clinically relevant prescribing omissions. The agreement between the two tools was only slight (κ = 0.093), indicating that they identify different subsets of inappropriate prescribing in the same patient population. These findings suggest that neither tool alone is sufficient for comprehensive medication review in Indian elderly patients. The observed low concordance also highlights the limited transferability of tools developed in Western populations to the Indian clinical setting. The present study supports the need for either the combined use of both criteria or the development of a context-specific screening tool tailored to Indian prescribing patterns and disease profiles to improve medication safety in older adults.

## Appendices

Case record form

https://drive.google.com/file/d/1VxTJHMD5Yij3LE_qV5-lIoIRkjXJLAqX/view?usp=drivesdk
